# Do skeletal muscle composition and gene expression as well as acute exercise-induced serum adaptations in older adults depend on fitness status?

**DOI:** 10.1186/s12877-021-02666-0

**Published:** 2021-12-15

**Authors:** Daniel A. Bizjak, Martina Zügel, Uwe Schumann, Mark A. Tully, Dhayana Dallmeier, Michael Denkinger, Jürgen M. Steinacker

**Affiliations:** 1 Division of Sports- and Rehabilitation Medicine, Department of Internal Medicine II, University of Ulm, Leimgrubenweg 14, 89075 Ulm, Germany; 2 Institute of Mental Health Sciences, School of Health Sciences, Ulster University, Newtownabbey, UK; 3 Agaplesion Bethesda Hospital, Geriatric Medicine Ulm University, Ulm, Germany; 4 Geriatric Center Ulm/Alb-Donau, Ulm, Germany; 5 Department of Epidemiology, Boston University School of Public Health, Boston, USA; 6 Department of Epidemiology and Medical Biometry, Ulm University, Ulm, Germany

**Keywords:** Physical fitness, Sedentary behavior, Health services for older individuals, Skeletal muscle, Molecular adaptations

## Abstract

**Background:**

Inactive physical behavior among the elderly is one risk factor for cardiovascular disease, immobility and increased all-cause mortality. We aimed to answer the question whether or not circulating and skeletal muscle biomarkers are differentially expressed depending on fitness status in a group of elderly individuals.

**Methods:**

Twenty-eight elderly individuals (73.36 ± 5.46 years) participated in this exploratory study after participating as part of the multinational SITLESS-clinical trial (implementation of self-management and exercise programs over 16 weeks). A cardiopulmonary exercise test (CPX) and resting skeletal muscle biopsy were performed to determine individual physiological performance capacity. Participants were categorized into a high physical fitness group (HPF) and a low physical fitness group (LPF) depending on peak oxygen uptake (VO_2_peak). Serum blood samples were taken before (pre) and after (post) CPX and were examined regarding serum BDNF, HSP70, Kynurenine, Irisin and Il-6 concentrations. Skeletal muscle tissue was analyzed by silver staining to determine the myosin heavy chain (MyHC) composition and selected genes by qRT-PCR.

**Results:**

HPF showed lower body weight and body fat, while skeletal muscle mass and oxygen uptake at the first ventilatory threshold (VO_2_T1) did not differ between groups. There were positive associations between VO_2_peak and VO_2_VT1 in HPF and LPF. MyHC isoform quantification revealed no differences between groups. qRT-PCR showed higher expression of *BDNF* and *BRCA1* in LPF skeletal muscle while there were no differences in other examined genes regarding energy metabolism. Basal serum concentrations of Irisin were higher in HPF compared to LPF with a trend towards higher values in BDNF and HSP70 in HPF. Increases in Il-6 in both groups were observed post.

**Conclusions:**

Although no association between muscle composition/VO_2_peak with fitness status in older people was detected, higher basal Irisin serum levels in HPF revealed slightly beneficial molecular serum and muscle adaptations.

**Trial registration:**

ClinicalTrials.gov, NCT02629666. Registered 19 November 2015.

**Supplementary Information:**

The online version contains supplementary material available at 10.1186/s12877-021-02666-0.

## Background

Sarcopenia is defined as the gradual loss of skeletal muscle mass, quality and strength during ageing and/or immobility [1]. Older people have a high prevalence of sarcopenia and altered skeletal muscle metabolism as a consequence of increased inactive lifestyle [2, 3]. Harvey et al. (2013) examined physical activity of older people in seven countries and found that approximately 67% of the age group > 60 years showed sedentary behavior of 8.5 h per day [4]. A systematic review of the amount of sedentary behavior in older people between 1981 and 2014 showed even higher values (average sedentary time of 9.4 h/day), equating to 65–80% of older people’s waking day [5]. Sarcopenia and increasing immobility is associated with a functional decline such as impaired gait speed, increased fall and hospitalization risk as well as a higher mortality rate [2, 6, 7]. Mechanistically, several pathways have been described. In addition to a downregulation of insulin sensitivity and cellular turnover, declining enzymatic activity in skeletal muscle [8, 9] has also been shown. Sarcopenic obesity, the combination of obesity and low muscle mass, is a serious health problem as it enhances the pro-inflammatory and adipokine response, promotes insulin resistance and is associated with increased strength loss and mortality [10].

This, however, can effectively be delayed by physical exercise [8, 11]. Several studies showed the beneficial effect of physical exercise in slowing down the loss of skeletal muscle mass and to increase life expectancy throughout different age groups [11, 12]. The beneficial effects of exercise training can be exerted at any time in life, regardless of the starting point [13, 14]. Exercise can also stimulate the expression of neurotrophic and angiogenic factors, reduce inflammation and thereby contributes to brain health and also reduce metabolic risk factors [15, 16].

The SITLESS project, a multicenter study conducted in four European countries (Spain, Denmark, Northern Ireland, Germany), addressed physical activity and sedentary behavior of community-dwelling elderly European citizens with a 16-week long training intervention [17]. In short, exercise referral schemes and self-management strategies were used to reduce sedentary behavior and improve health, quality of life and physical function, as well as psychosocial outcomes in the long term. In a sub-study, we aimed to test the hypothesis that fitter older individuals more likely have lower inflammation, improved cellular regulation processes involved in regeneration, metabolism and genomic health and a different muscle structure than unfit age-matched controls. Skeletal muscle biopsy samples at rest and blood samples before and after an acute exercise test were taken from fit and unfit study participants to analyze ageing and performance markers on the RNA and protein level for possible future diagnostic applications.

## Materials and methods

**Fig. 1:**
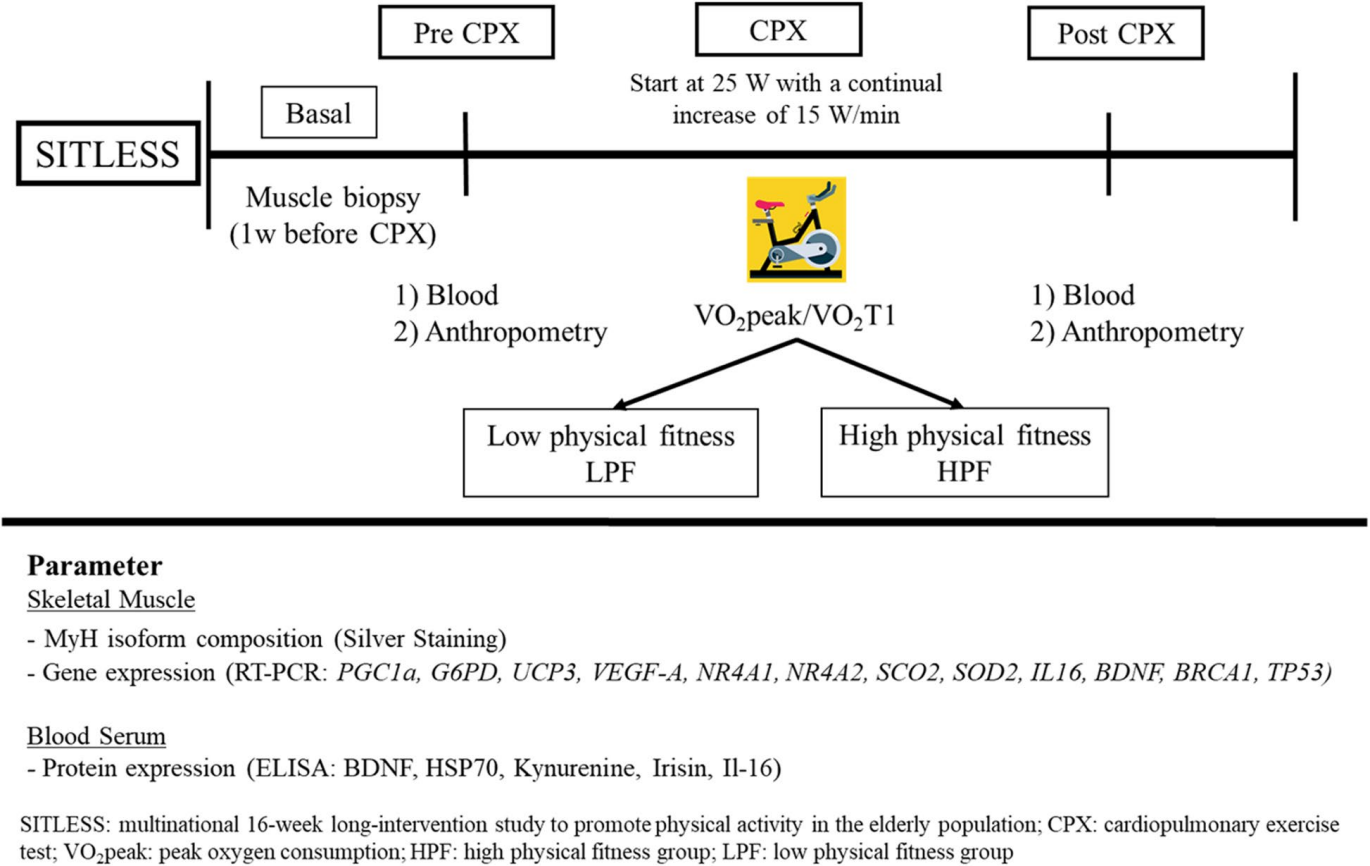
Schematic overview of the study design. After SITLESS, participants were asked to participate in this sub-study. A muscle biopsy was conducted at least one week before the CPX. Muscle samples were used for Myosin-Heavy chain (MyH) isoform composition and gene expression analysis. Blood was sampled before (pre) and directly after the CPX (post), and serum protein expression determined. Participants were classified according to the VO_2_peak measured during CPX by spiroergometry into HPF and LPF. (SITLESS: multinational 16-week long-intervention study to promote physical activity in the elderly population; CPX: cardiopulmonary exercise test; VO_2_peak: peak oxygen consumption; HPF: high physical fitness group; LPF: low physical fitness group)

A subgroup of the original SITLESS clinical trial [17] with residence in Germany was recruited to participate in this exploratory study and attended two additional visits at the Division of Sports- and Rehabilitation Medicine at Ulm University (Germany) after the end of the 16-week long SITLESS intervention study [18, 19]. Participants (16 female, 12 male) were divided into two groups depending on their peak oxygen consumption (VO_2_peak/kg): 1) Individuals with low physical fitness, (LPF, VO_2_peak/kg female ≤ 17 ml/min/kg, male ≤ 23 ml/min/kg); and 2) individuals with high physical fitness (HPF, VO_2_peak/kg female ≥ 18 ml/min/kg, male ≥ 24 ml/min/kg). As VO_2_peak represents the aerobic fitness status at higher intensities [20], we calculated the median for each woman and men and dichotomized the groups in HPF and LPF according to the higher and lower percentiles around these values. A graphical representation of the study design is provided in Fig. 1.

Inclusion criteria were: aged ≥65 years; male or female; able to perform the physical exercise training and to walk a distance of at least 400 m by themselves; and previously physically inactive (< 150 min moderate exercise or < 75 min vigorous exercise per week). Dementia or other malignant progressive cardiovascular or oncologic diseases led to study exclusion. Detailed exclusion criteria are listed in Additional File 1.

This pilot study was approved by the Ethical Review Committee of Ulm University (No. 354/15). It conforms with the Declaration of Helsinki and all participants gave written informed consent to participate prior to taking part in this study.

### Body composition and performance testing

Body composition was determined by body-impedance-analysis (BIA, InBody 770, Eschborn, Germany). Performance and aerobic capacity were determined by a spiroergometric cardiopulmonary exercise test (CPX). An incremental test on a bicycle ergometer (Lode Excalibur Sport, Groningen, The Netherlands) was performed to measure the first ventilatory threshold (VO_2_VT1) as well as peak oxygen consumption (VO_2_peak). After 2 min of unloaded pedaling, the incremental test started at 25 Watt (W) with a continual increase of 15 W/min. The test was aborted if an individual reached physical exhaustion or medical indications prevailed. A dynamic mixing chamber was used to determine breathing gases (Cortex MetaMax 3X, Leipzig, Germany). Calculation of the ventilatory thresholds were conducted by the V-slope method of Schneider et al. [21].

### Blood analysis

Twenty milliliter of blood was drawn directly before (pre) and after (post) the CPX from the antecubital vein to determine adaptations of acute exercise on the molecular level. Blood was sampled in serum vacutainers, coagulated for at least 30 min in an upright position and centrifuged for 10 min at 3800 rpm. Serum was stored at − 80 °C until further analysis.

Concentrations of brain-derived neurotrophic factor (BDNF, R&D Systems, Minneapolis, USA), Interleukin 6 (Il-6, R&D Systems, Minneapolis, USA), Irisin (Phoenix Pharmaceuticals, California, USA) and Heat shock protein 70 (HSP70, Enzo Life Sciences GmbH, Lörrach, Germany) were analyzed by ELISAs according to the respective manufacturer’s instruction.

Kynurenine was determined by the following protocol: 1000 μM of L-kynurenine sulfate salt (Sigma-Aldrich, Munich, Germany) was diluted 1:20 with 15% Trichloric acid (TCA) to a final concentration of 50 μM. This stock solution was further diluted with TCA to 25 μM, 12.5 μM, 6.25 μM, 3.125 μM, 1.56 μM and 0.78 μM for standard curve determination. Aqua dest. Was used as blank solution. One fifty microliter serum sample was mixed with 100 μl of 30% TCA and centrifuged at 20000 g for 10 min and 4 °C. One fifty microliter of standard solutions and blank as well as 150 μl of the supernatant were pipetted into a 96 well plate in duplicates and incubated for 15 min at 65 °C. 0.15 g of diaminobenzoic acid (DABA) was diluted in 10 ml 100% acetic acid and 150 μl of this final solution was mixed with samples for 5 min at RT. Fluorescence was measured with a plate reader and the concentration of samples calculated by the slope of the linear regression line of the optical density from 492 to 620 nm.

### Muscle biopsies

The skeletal muscle biopsy took place 1-7 days before the CPX using the Bergström technique according to published protocols [22]. In detail, participants were encouraged to perform their regular daily behavior after the end of SITLESS. The day before and on the day of the muscle biopsy, all participants were asked to refrain from strenuous physical exercise. A careful medical examination including medical history, medication and ECG was obtained and all examinations were performed between 1:30–3 pm to minimize inter-individual effects. When the participant was suitable for biopsy, skeletal muscle samples will be obtained under local anesthesia from the *Musculus vastus lateralis* (femur) of the dominant side of the subjects (or the healthy leg, respectively) approximately 20 cm above the knee. The Bergström-Needle with 4 mm outer diameter allows attaining of 100–200 mg muscle tissue.

For RNA isolation, muscle tissue was incubated for 24 h with RNAlater (QIAGEN GmbH, Hilden, Germany) at 4°C and then stored in cryotubes at − 80°C until further analysis. For protein examination, muscle tissue was immediately cryopreserved with liquid nitrogen and stored at − 80°C until further analysis.

The biopsy site was closed with suture tape and slight compression was applied using a flexible bandage. Participants could walk and train at the same day and were fully able to use their legs.

### Protein preparation and silver staining

To determine the respective protein myosin heavy chain (MHC) isoform abundance, muscle samples were incubated with 250 μl Pierce™ RIPA buffer (Thermo Fisher Scientific, MA, USA), mixed with cOmplete™ Mini EDTA-free Protease Inhibitor Cocktail (Roche, Basel, Switzerland), homogenized and incubated for 10 min on ice in Pierce™ RIPA buffer to a final concentration of 5 μg/μl muscle protein. After centrifugation (4°C, 14000 rpm, 20 min), the supernatant was used for SDS-PAGE according to the manufacturer’s instructions (Mini-Protean TGX Gels 4–20%, BIO-RAD, Berkeley, USA). Afterwards, silver staining was performed with the Silver Stain kit (BIO-RAD, #161–0443, Berkeley, USA) according to the manufacturer’s instructions. The relative protein abundance was analyzed with Image J 1.51 (ImageJ, NIH, Maryland, USA).

### RT-PCR

Muscle biopsy samples of HPF and LPF were examined regarding gene expression of genes involved in aerobic metabolism, anti-oxidative response, aging and tumor development. Targets were *PGC1a, G6PD, UCP3, VEGFa, NR4A1, NR4A2, SCO2, SOD2, IL16, BDNF, BRCA1* and *TP53*. RNA was quantified with spectrophotometry (NanoDrop 2000c, Thermo Scientific, Massachusetts, USA) and transcribed to cDNA with the QantiTect® Reverse Transcription Kit (QIAGEN GmbH, Hilden, Germany) according to the manufacturer’s instructions. cDNA was used to determine the expression with Real Time qPCR (RT-qPCR) analogous to established protocols [23] with GAPDH as established reference gene for endurance exercise [23, 24].

### Statistical analysis

Statistical analysis was performed using GraphPad PRISM (Version 9, La Jolla, USA). Data were tested for Gaussian distribution. Normally distributed data of blood variables were analyzed for inter- and intra-group differences with a 2-way ANOVA followed by Tukey Post-hoc test. Otherwise, a mixed model analysis was used followed by Dunn’s Post-hoc test. To determine associations between skeletal muscle mass (SMM), age, VO_2_peak and VO_2_T1, Spearman correlation analyses followed by two-tailored t-test were performed to determine statistical significance. Descriptive statistics of the data are presented as mean ± standard deviation. Statistical differences were considered to be significant for values of *p* ≤ 0.05.

## Results

### Physical performance measures and anthropometry

The study population consisted of 28 participants (14 HPF, 14 LPF). A detailed anthropometric description is provided in Table 1.

**Table 1:** Anthropometric data of all participants and subdivided into trained (HPF) and untrained control group (LPF)

Variables	Total (***n*** = 28)		HPF (***n*** = 14)		LPF (***n*** = 14)	
	Mean	SD	Mean	SD	Mean	SD
**Age [y]**	75.25	5.44	74.4	5.7	76.1	5.2
**Height [cm]**	167.02	9.22	166.7	9.8	167.3	8.9
**Body mass [kg]**	79.96	18.34	71.54 *	14.69	88.41	18.22
**BMI [kg/m**^**2**^**]**	28.22	5.05	25.51 **	3.13	30.93	5.23
**Skeletal muscle mass [kg]**	28.47	6.23	28.04	6.13	28.90	6.53
**Body fat mass [kg]**	27.80	11.50	20.20 ***	6.79	35.40	10.14
**Body fat [%]**	33.80	8.80	27.80 ***	6.18	39.70	6.80
**Skeletal muscle mass/Body surface area [kg/m**^**2**^**]**	15.00	1.71	15.40	1.62	14.80	1.72
	*N* = 26		*N* = 14		*N* = 12	
**VO**_**2**_**peak/kg [l/min/kg]**	21.25	5.586	24.71 ***	4.79	17.20	3.26
**VO**_**2**_**VT1 [l/min]**	1.04	0.35	1.10	0.37	0.97	0.32

*p ≤ 0.05

**p ≤ 0.01

***p ≤ 0.001 indicate inter-group differences

**Fig. 2:**
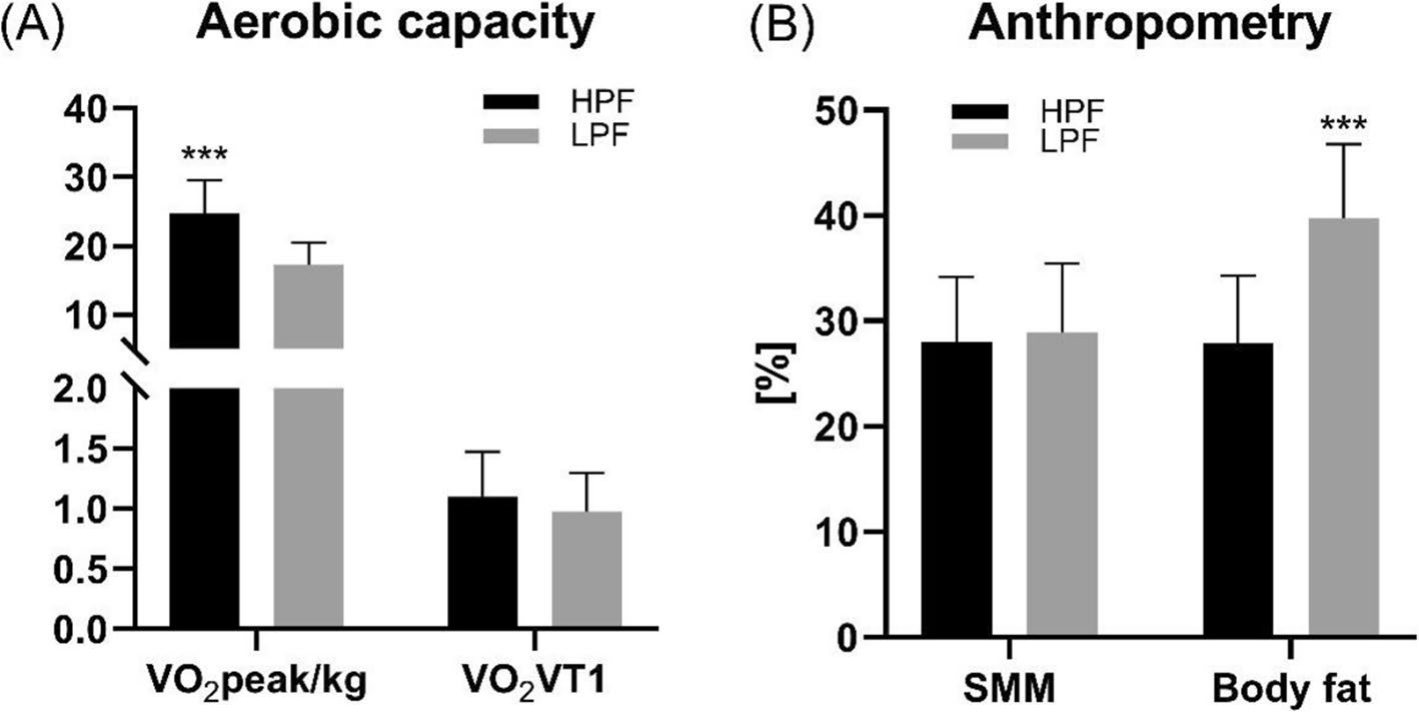
Differences in aerobic capacity and anthropometry in HPF and LPF. **A** HPF showed significantly higher VO_2_peak/kg values compared with LPF (*p* < 0.001) with no difference in first ventilatory threshold VO_2_VT1. **B** Skeletal muscle mass (SSM) was statistically not different between the groups while body fat was higher in LPF (p < 0.001)

HPF participants had lower body mass (*p* = 0.0119), BMI (*p* = 0.0026) and body fat mass (p < 0.001) (Table 1) as well as lower percentage body fat (*p* < 0.001) and higher VO_2_peak (p < 0.001) compared to LPF (Fig. 2). Examination of the respiratory quotient (RQ) at maximal exertion showed a mean of 1.10 +/− 0.09, which is clear sign of objective exhaustion in all subjects. Gender analysis revealed no significant differences in the fat parameters or VO_2_peak between female and male participants. Only VO_2_T1 was significantly higher in males vs. females (*p* = 0.0003) (Additional File 1). As the results were based on the fitness level determined by VO_2_peak, no gender effect is assumed to bias the data.

#### Significant positive correlation between VO2peak and VO2VT1 in HPF and LPF

**Fig. 3:**
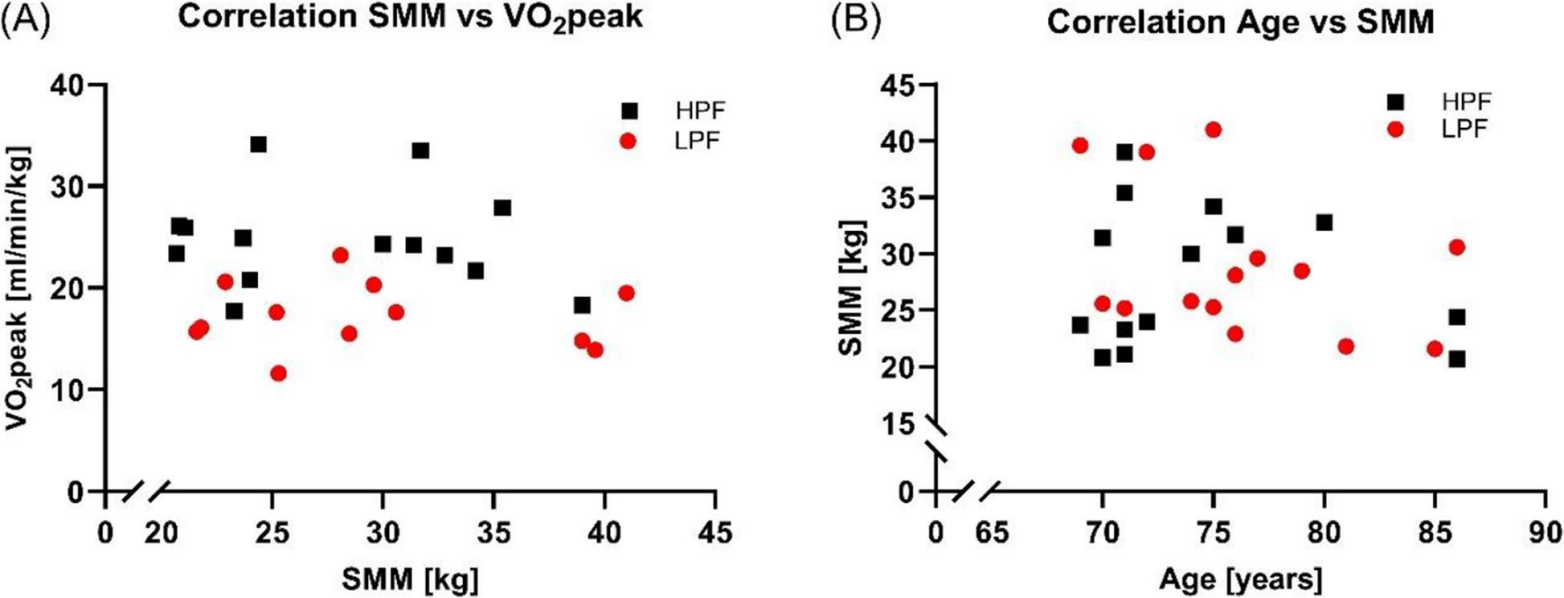
Correlation of variables known to affect performance, and which are changing during ageing. **A** Correlation of skeletal muscle mass (SMM) and VO_2_peak revealed no association in HPF or LPF. **B** No association was observed regarding age and SMM in both groups

Although there was no correlation between SMM, age, VO_2_peak and VO_2_T1 (Fig. 3), a positive correlation between VO_2_peak and VO_2_VT1 in HPF (*p* = 0.009) and LPF (*p* = 0.004) was observed (Additional File 1). A separate analysis showed no effect of sex on the tested parameters. In female participants, there were negative correlations seen between body fat percentage and VO_2_peak (*p* = 0.003) and body fat mass and age (0.022) (Additional File 1).

#### RT-PCR revealed no differences except for BDNF and BRCA1

Analysis of selected genes involved in aerobic metabolism, anti-oxidative response, ageing, and tumor development showed no difference in the expression between HPF and LPF, except for BDNF and BRCA1 (Additional File 2). Except for a limited number of PCR-results for BDNF (HPF *n* = 5, LPF *n* = 6), all targets could be reliable detected.

#### Myosin heavy chain (MyHC) composition did not differ between HPF (*n* = 13) and LPF (*n* = 12)

**Fig. 4:**
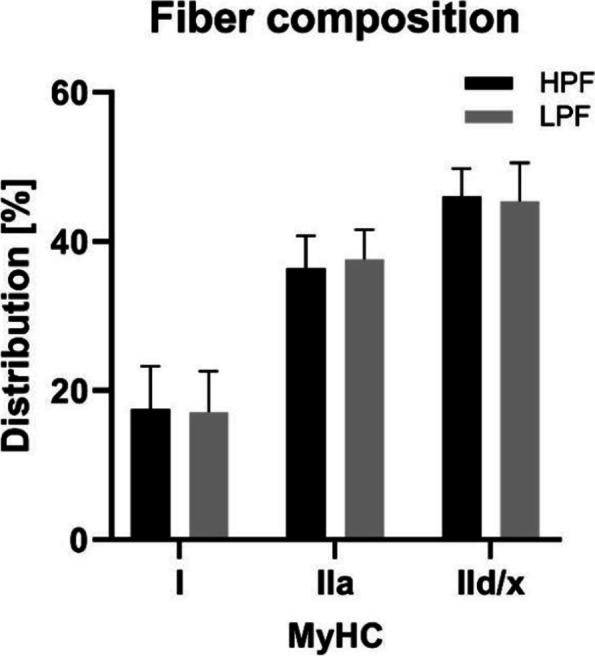
MyHC fiber type composition of HPF and LPF. No differences were observed either in Type I fibers (HPF 17.53 ± 5.76%; LPF = 17.06 ± 5.55%), Typ IIa (HPF 36.43 ± 4.34%; LPF = 37.58 ± 4.02%) or Type IId/x fibers (HPF 46.03 ± 3.76%; LPF = 45.37 ± 5.14%)

Both groups showed highest fiber type percentage in MyHC IId/x fibers, followed by MyHC IIa and MyHC I fibers (Fig. 4).

#### Serum protein quantification of selected factors revealed significant basal differences in Irisin and adaptations in Il-6 response in both groups

**Fig. 5:**
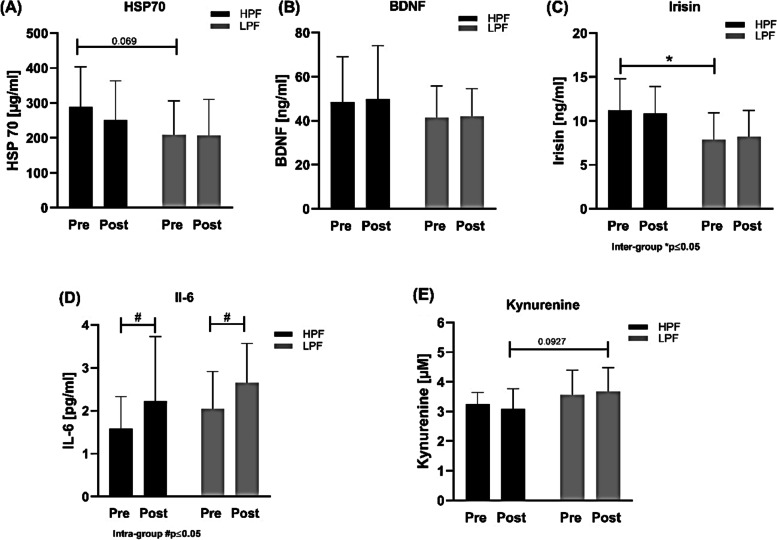
Changes in serum concentrations of molecules involved in ageing, muscle damage and neurotrophic adaptation. HPF and LPF were examined after an acute exercise test (pre vs. post). **A** HSP70 showed non-significant higher basal values in HPF (*p* = 0.069) compared to LPF. **B** Plasma levels of BDNF were non-significantly higher in HPF pre and post. **C** Irisin concentrations were pre higher in HPF compared to LPF, but this difference was blunted post. **D** Serum concentration of inflammation marker Il-6 increased post in HPF and LPF. **E** Kynurenine did not differ in both groups, but due to an increase in LPF and a decrease in HPF post a tendency for different adaptations (*p* = 0.0927) was observed

Serum samples were taken before (pre) and after (post) an acute exercise test on a bicycle ergometer for analysis of HSP70, BDNF, Irisin, Il-6 and kynurenine protein concentration (Fig. 5). Subsequent ELISA analysis showed significant differences between HPF and LPF in basal values of irisin (*p* = 0.0195) (Fig. 5C). There was an increase in HPF (*p* = 0.0124) and LPF (*p* = 0.0146) after exercise in Il-6 (Fig. 5D). Kynurenine (*p* = 0.0927) and HSP70 (*p* = 0.069) only showed post a tendency for inter-group changes (Fig. 5E).

## Discussion

In this pilot study we found that although maximal oxygen uptake and body composition of fitter older adults were higher compared to unfit peers, most of the basal gene expression related to ageing, performance, and associated metabolism as well as fiber type composition did not significantly differ between HPF and LPF. On the other hand, slight differences were observed regarding protein concentrations before and after an acute exercise test.

### Performance and body/muscle composition

Several studies have assessed sedentary behavior and physical inactivity in older adults taking into account differing nationalities, social/educational level and sex [4, 25–27], and uniformly observed a tendency for increasing inactive physical behavior among the elderly population. Although the popular allusion that “sitting is the new smoking” is scientifically not sound [28, 29], an inactive lifestyle, especially in older people, increases the risk for cardiovascular diseases, loss of skeletal muscle mass and malfunction as well as all-cause mortality [7, 13, 28].

The combination of increasing age and loss of skeletal muscle mass and/or functionality is associated with decreased aerobic capacity, shown by decreased VO_2_max [30]. The examination of our fitter HPF and unfit LPF showed distinct differences in performance and body composition. Especially the workload at higher intensities could be better maintained by HPF, seen by higher VO_2_peak/kg, whereas there seemed no difference between groups during moderate intensities at VO_2_VT1. Maintaining a higher VO_2_peak during life can reduce the risk of multiple comorbidities, all-cause mortality, and loss of independence during aging [31].

The aging-associated capillarization and aerobic enzyme activity decline is equally slowed down by lifelong exercise, which can have a direct beneficial influence on cardiovascular health in women and men [32]. In our mostly inactive LPF participants, the body fat percentage was significantly higher. It is well known that a high body fat percentage contributes to increased risk of developing cardiovascular diseases, diabetes and immobility [33]. On the other hand, several studies have shown that weight loss intervention with a combination of diet and exercise improves muscle strength and muscle quality in addition to fat loss among older obese people and consequently attenuates sarcopenic symptoms and pro-inflammatory responses [10]. On the muscular level, increasing age and immobility lead to alterations in the MyHC isoform distribution, fiber type composition and fiber activation [2, 12, 34]. Interestingly, we did not detect any correlation between skeletal muscle mass, VO_2_peak and age in both groups and, what is more, skeletal muscle mass as well as MyHC composition were not different between HPF and LPF.

Fiber size was not assessed, which may have given further insights into muscle adaptations and decline as muscle strength is a strong predictor of severe mobility limitation, slow gait speed, increased fall risk, risk of hospitalization, and high mortality rate [2, 34–36]. For example, Miljkovic et al. showed that elderly adults with low muscle strength have a 2.6-fold greater risk of severe mobility limitation, 4.3-fold greater risk for slow gait speed, and 2.1-fold greater risk of mortality compared to elderly adults with high muscle strength [2].

### Skeletal muscle gene expression

Beside adaptations in fiber type morphology, regular exercise can slow down age-related alterations on the molecular skeletal muscle level with beneficial changes in oxidative enzyme activity, DNA repair and skeletal neurotrophic factors like BDNF [11, 12, 37]. Skeletal muscular expression analysis of genes involved in gene stability, energy metabolism and mitochondrial activity (*PGC1a, G6PD, UCP3, VEGFa, NR4A1, NR4A2, SCO2, SOD2, IL16,* and *TP53*) revealed no difference in HPF and LPF regarding energy metabolism or mitochondrial activity. Significant differences could only be observed in *BDNF* and *BRCA1* levels with higher basal expression in LPF. The latter contrasts with previous studies, which showed increased expression in *BRCA1*, responsible for genomic stability, with regular exercise [38, 39]. We observed a high intra-group variability in the expression profiles which may contribute to this result. A further possible explanation may be the known blunted response on the protein level in older people [9].

### Serum protein adaptations

Although a higher basal *BDNF* skeletal muscle mRNA expression was seen for LPF, serum protein concentrations showed non-significant elevated levels in HPF pre and post exercise compared to LPF. Until now, skeletal muscle expression of BDNF in older people after exercise is incompletely understood and has been rarely examined: accumulating evidence on circulating protein levels seem to hint to transiently up-regulation of BDNF concentration after acute exercise, but with no long-term changes with regular training [40–42]. Therefore, BDNF may not be differently regulated depending on physical fitness status in older individuals and similar levels might be expected.

Beside BDNF, Küster et al. evaluated Irisin and the kynurenine pathway as novel blood-based biomarkers for physical and cognitive performance [42]. Ten weeks of cognitive and physical exercise training in older people were associated with potentially beneficial decreased kynurenine metabolites, whereas no significant changes in Irisin levels could be observed, which is in contrast to other studies [43]. The main function of the myokine Irisin is thermogenesis regulation by hormonal inducement of increasing energy expenditure, promoting weight loss, and decreasing insulin resistance [44]. Irisin is also a potential circulating mediator of exercise which benefits brain health and neuroprotection, as it was shown that it is secreted upon exercise in mice and humans, where it apparently promotes BDNF release [16]. Furthermore, the PGC-1*α*-FNDC5/Irisin-BDNF-signaling pathway has been proposed for resistance exercise that increases the expression of FNDC5/Irisin and in turn induces BNDF with subsequently beneficial adaptations in the transcription and transport of mRNA along dendrites, growth, differentiation, and survival of neurons [44].

In our study, significantly higher serum baseline values of circulating Irisin in HPF compared to LPF were found. Circulating Irisin levels were shown to be negatively correlated with cardiometabolic risk in sedentary participants, with an active lifestyle positively increasing Irisin levels [15]. This is in line with higher VO_2_peak data of HPF, which implies a higher general fitness of this sub-group. For serum kynurenine, only a trend for decreased concentrations in HPF and increased concentration in LPF was observed post. This might suggest better coping capacity with the physical exercise test, as elevated kynurenine levels were associated during and after stressful cognitive and physical periods [42].

Serum HSP70 concentration did not significantly differ between HPF and LPF. The versatile heat shock protein HSP70 serves as a molecular chaperone involved in many cellular processes [45]. It has a profound impact on protein turnover, energy metabolism, muscle function/regeneration, hypertrophy and adaptation, while sarcopenia leads to decreased HSP70 content due to muscle loss [46]. Furthermore, a blunted expression of HSP70 in skeletal muscle in response to chronic exercise during aging was observed [9, 46], which may support our observation of unchanged HSP70 response in both HPF and LPF.

The proinflammatory cytokine Il-6 is induced after stressful events like intensive exercise, but has the additional function as an anti-inflammatory myokine [47]. It is one of the key molecules which controls the catabolic effect in myoblasts through inhibition of myogenesis and protein synthesis [48]. Higher plasma levels of Il-6 are predominately found in older people and were associated with lower muscle mass and muscle strength [48], which may be driven by the chronic low-grade inflammatory status and contributes to muscular functional impairment [8]. Il-6 serum basal levels of HPF and LPF were not significantly different and both groups showed increased values after the exercise test. This observation indicates a similar inflammation process regardless of physical fitness status, which may modulate the so called “inflammageing” in older people [8]. A third sample time point after the exercise test, e.g. 1 or 2 h later, may have given further insights in the long-term process of the Il-6 regulation. Short-term increases of inflammatory cytokines are common responses to exercise, whereas prolonged increases have harmful consequences.

### Limitations

In this pilot-study, physically fit and unfit older men and women performed an acute exercise test with pre and post blood sampling, but a muscle biopsy sample was only taken at rest and so restricted to one time point. The relatively small participant number and muscle sample amount limits to a certain extent the genetic profiling, muscle protein analysis possibility and thus definitive conclusions thereof due to high interindividual expression. Nevertheless, the examined rare muscle biopsy samples in this susceptible older population give valuable insights into structural and adaptive processes in the ageing musculature.

## Conclusions

Valenzuela et al. stated that “The sooner the better, but never too late” in their seminal review regarding exercise adaptations and health effects in the older population [14]. Serum molecular adaptations in older individuals to an acute exercise test seem to depend on fitness level. Although we did not detect a significant association between basal muscle parameters like SSM or fiber type composition and VO_2_peak/kg with fitness status, higher basal levels of Irisin in HPF revealed slightly beneficial long-term adaptations due to higher fitness level but most adaptations may be blunted during ageing. A significant lower inflammation after the acute test or a different muscle composition of fit- compared to unfit age-matched subjects could not be detected, suggesting that probably a still higher fitness status than our previously unactive participants has to be maintained to gain beneficial long-term adaptations in the older population.

## Supplementary Information

**Additional file 1. **Detailed inclusion and exclusion criteria of study participants. **Table a.** Descriptive statistics of body fat mass (kg), % body fat, relative VO_2_ peak, VO_2_VT1 for males and females. **Table b.** Values subdivided to the respective male and female participants. **Table c:** *P*-values after Spearman correlation analysis for aerobic parameters VO_2_peak and VO_2_VT1, age and skeletal muscle mass SMM in the respective HPF and LPF. **Figure a.** Spearman correlation matrix with respective r-value after analysis of age, maximal oxygen consumption VO_2_peak/kg, first ventilatory threshold VO_2_VT1 and skeletal muscle mass SMM in HPF and LPF. **Figure b.** Spearman correlation matrix with respective r-value after analysis of age, maximal oxygen consumption VO_2_peak/kg, first ventilatory threshold VO_2_VT1 and skeletal muscle mass SMM in female and male participants.

**Additional file 2: Figure c.** Violin plot of Relative Quantification (RQ) of selected gene expression related to ageing, performance and associated metabolism. Except for *BDNF* (*p* = 0.0303) and *BRCA1* (*p* = 0.0214), no difference was observed between HPF and LPF. **Figure d:** Correlation matrix and respective Spearman’s r for VO_2_peak of HPF vs gene expression. **Table d.** Relative Quantification (RQ) of selected gene expression related to ageing, performance and associated metabolism. Except for *BDNF* (*p* = 0.0303) and *BRCA1* (*p* = 0.0214), no difference was observed between HPF and LPF. * *p* ≤ 0.05 indicate inter-group differences. **Table e.** Mean MyHC composition as well as MyHC composition for HPF and LPF with regard to MyHC I, MyHC IIa and MyHC IId/x fiber type.

## Data Availability

All pseudonymized data which are not included in the additional files or the manuscript are available on reasonable request from the corresponding author.

## Abbreviations

BDNF: Brain-derived neurotrophic factor
BRCA1: Breast cancer gene 1
BMI: Body mass index
CPX: Cardiopulmonary exercise test
HPF: High physical fitness group
HSP70: Heat-shock protein 70
Il-6: Interleukin 6
LPF: Low physical fitness group
MyHC: Myosin heavy chain
RQ: Respiratory quotient
SMM: Skeletal muscle mass
VO_2_peak: Peak oxygen uptake
VO_2_T1: Oxygen uptake at the first ventilatory threshold
