# Supplementary material for: Do skeletal muscle composition and gene expression as well as acute exercise-induced serum adaptations in older adults depend on fitness status?
Source: BMC Geriatr. 2021 Dec 15;21:697. doi: 10.1186/s12877-021-02666-0 (PMC8672635; doi:10.1186/s12877-021-02666-0)
Supplement: Supplementary file 1 — Additional file 1. Detailed inclusion and exclusion criteria of study participants. Table a. Descriptive statistics of body fat mass (kg), % body fat, relative VO2 peak, VO2VT1 for males and females. Table b. Values subdivided to the respective male and female participants. Table c: P-values after Spearman correlation analysis for aerobic parameters VO2peak and VO2VT1, age and skeletal muscle mass SMM in the respective HPF and LPF. Figure a. Spearman correlation matrix with respective r-value after analysis of age, maximal oxygen consumption VO2peak/kg, first ventilatory threshold VO2VT1 and skeletal muscle mass SMM in HPF and LPF. Figure b. Spearman correlation matrix with respective r-value after analysis of age, maximal oxygen consumption VO2peak/kg, first ventilatory threshold VO2VT1 and skeletal muscle mass SMM in female and male participants. [file 12877_2021_2666_MOESM1_ESM.docx]

Additional File 1

**Detailed inclusion and exclusion criteria of study participants**

**Inclusion:**

- Age ≥65 years
- Gender male or female
- Performance:
- => able to perform the physical exercise training and to walk a distance of at least 400 m by themselves
- => previously physically inactive (<150 min moderate exercise or <75 min vigorous exercise per week)
- Participant of the SITLESS-clinical trial

**Exclusion:**

- Dementia or other malignant progressive cardiovascular or oncologic diseases
- Disorders of bleeding, anticoagulant therapy, malignant hypertension
- Heart failure clinical state III or IV (NYHA criteria), angina pectoris in daily life or any signs of significant cardiac ischemia during exercise
- Cardiac arrhythmia and severe coordination disturbances
- Hemodynamically relevant cardiac valve disease, and other severe diseases, e.g. liver cirrhosis or end stage renal disease

**Table a. Descriptive statistics of body fat mass (kg), % body fat, relative VO_2_ peak, VO_2_VT1 for males and females.** Due to illness, there were two missing participants in the CPX test after basal muscle biopsy and screening.

|  | **Body Fat mass Female [kg]** | **Body Fat mass Male [kg]** | **% Body Fat Female** | **% Body Fat Male** |
| --- | --- | --- | --- | --- |
| **Number of participants** | 16 | 12 | 16 | 12 |
|  |  |  |  |  |
| **Mean** | 26.68 | 29.22 | 35.78 | 31.11 |
| **SD** | 12.72 | 10.59 | 10.26 | 6.330 |
| **Std. Error of Mean** | 3.180 | 3.058 | 2.565 | 1.827 |
| **p-value (f vs m)** | 0.5806  ns | | 0.1768  ns | |
|  |  | |  | |
|  | **VO_2_peak/kg Male** | **VO_2_peak/kg Female** | **VO_2_VT1 Male** | **VO_2_VT1 Female** |
| **Number of participants** | 11 | 15 | 11 | 15 |
|  |  | |  | |
| **Minimum** | 16.10 | 11.60 | 0.9100 | 0.5400 |
| **25% Percentile** | 20.30 | 15.50 | 1.080 | 0.7100 |
| **Median** | 23.40 | 17.70 | 1.150 | 0.8500 |
| **75% Percentile** | 26.10 | 23.20 | 1.550 | 0.9600 |
| **Maximum** | 33.50 | 34.10 | 2.120 | 1.150 |
| **Range** | 17.40 | 22.50 | 1.210 | 0.6100 |
|  |  |  |  |  |
| **Mean** | 23.62 | 19.51 | 1.302 | 0.8480 |
| **Std. Deviation** | 4.670 | 5.699 | 0.3714 | 0.1640 |
| **Std. Error of Mean** | 1.408 | 1.471 | 0.1120 | 0.04234 |
| **p-value (f vs m)** | 0.0622  ns | | 0.0003  *** | |

**Table b:** Values subdivided to the respective male and female participants

| **Variables** | Female (n=16) | | Male (n=12) | |
| --- | --- | --- | --- | --- |
|  | Mean | SD | Mean | SD |
| **Age [y]** | 75.38 | 5.78 | 74.92 | 4.89 |
| **Height [cm]** | 160.83 | 6.24 | 175.28 | 4.46 |
| **Body mass [kg]** | 71.38 | 14.50 | 91.4 | 15.75 |
| **BMI [kg/m²]** | 27.15 | 5.00 | 29.64 | 4.51 |
| **Skeletal muscle mass [kg]** | 24.00 | 2.55 | 34.40 | 4.13 |
| **Body fat mass [kg]** | 26.70 | 12.32 | 29.20 | 10.14 |
| **Body fat [%]** | 35.80 | 9.93 | 31.1 | 6.06 |
| **Skeletal muscle mass/Body surface area [kg/m²]** | 13.80 | 1.07 | 16.60 | 0.85 |
|  | N=11 | | N=15 | |
| **VO_2_peak/kg [l/min/kg]** | 19.51 | 5.70 | 23.62 | 4.67 |
| **VO_2_VT1 [l/min]** | 0.848 | 0.16 | 1.30 | 0.37 |

**BMI: Body mass index; VO_2_peak/kg: peak oxygen uptake relative to body mass; VO_2_VT1: first ventilatory threshold**

**Table c:** P-values after Spearman correlation analysis for aerobic parameters VO_2_peak and VO_2_VT1, age and skeletal muscle mass SMM in the respective HPF and LPF.

| **Variables** | **VO_2_peak/kg**  **HPF** | **VO_2_peak/kg**  **LPF** | **VO_2_VT1**  **HPF** | **VO_2_VT1**  **LPF** | **Age**  **HPF** | **Age**  **LPF** | **SSM**  **HPF** | **SSM**  **LPF** |
| --- | --- | --- | --- | --- | --- | --- | --- | --- |
| **VO_2_peak/kg**  **HPF** |  | 0.822 | **0.009** | 0.345 | 0.845 | 0.607 | 0.762 | 0.097 |
| **VO_2_peak/kg**  **LPF** | 0.822 |  | 0.822 | **0.004** | 0.019 | 0.443 | 0.943 | 0.754 |
| **VO_2_VT1**  **HPF** | **0.009** | 0.822 |  | 0.812 | 0.910 | 0.592 | 0.829 | 0.001 |
| **VO_2_VT1**  **LPF** | 0.345 | **0.004** | 0.812 |  | 0.044 | 0.236 | 0.584 | 0.812 |
| **Age**  **HPF** | 0.845 | 0.019 | 0.910 | 0.044 |  | 0.046 | 0.738 | 0.336 |
| **Age**  **LPF** | 0.607 | 0.443 | 0.592 | 0.236 | 0.046 |  | 0.575 | 0.354 |
| **SMM**  **HPF** | 0.762 | 0.943 | 0.829 | 0.584 | 0.738 | 0.575 |  | 0.221 |
| **SMM**  **LPF** | 0.097 | 0.754 | 0.001 | 0.812 | 0.336 | 0.354 | 0.221 |  |

**SMM: Skeletal muscle mass; VO_2_peak/kg: peak oxygen uptake relative to body mass; VO_2_VT1: first ventilatory threshold**

**Figure a:** Spearman correlation matrix with respective r-value after analysis of age, maximal oxygen consumption VO_2_peak/kg, first ventilatory threshold VO_2_VT1 and skeletal muscle mass SMM in HPF and LPF.

**Figure b:** Spearman correlation matrix with respective r-value after analysis of age, maximal oxygen consumption VO_2_peak/kg, first ventilatory threshold VO_2_VT1 and skeletal muscle mass SMM in female and male participants.
