# Supplementary material for: Do skeletal muscle composition and gene expression as well as acute exercise-induced serum adaptations in older adults depend on fitness status?
Source: BMC Geriatr. 2021 Dec 15;21:697. doi: 10.1186/s12877-021-02666-0 (PMC8672635; doi:10.1186/s12877-021-02666-0)
Supplement: Supplementary file 2 — Additional file 2: Figure c. Violin plot of Relative Quantification (RQ) of selected gene expression related to ageing, performance and associated metabolism. Except for BDNF (p = 0.0303) and BRCA1 (p = 0.0214), no difference was observed between HPF and LPF. Figure d: Correlation matrix and respective Spearman’s r for VO2peak of HPF vs gene expression. Table d. Relative Quantification (RQ) of selected gene expression related to ageing, performance and associated metabolism. Except for BDNF (p = 0.0303) and BRCA1 (p = 0.0214), no difference was observed between HPF and LPF. * p ≤ 0.05 indicate inter-group differences. Table e. Mean MyHC composition as well as MyHC composition for HPF and LPF with regard to MyHC I, MyHC IIa and MyHC IId/x fiber type. [file 12877_2021_2666_MOESM2_ESM.docx]

**Additional File 2**

**Figure c: Violin plot of Relative Quantification (RQ) of selected gene expression related to ageing, performance and associated metabolism.** Except for *BDNF* (p=0.0303) and *BRCA1* (p=0.0214), no difference was observed between HPF and LPF.

**Figure d: Correlation matrix and respective Spearman’s r for VO2peak of HPF vs gene expression**

**Table d: Relative Quantification (RQ) of selected gene expression related to ageing, performance and associated metabolism.** Except for *BDNF* (p=0.0303) and *BRCA1* (p=0.0214), no difference was observed between HPF and LPF. * p ≤ 0.05 indicate inter-group differences.

| **Variables** | **HPF (n=14)** | | | **LPF (n=14)** | | |
| --- | --- | --- | --- | --- | --- | --- |
|  | **Mean** | **SD** | **n** | **Mean** | **SD** | **n** |
| ***BDNF*** | 0.46 | 0.27 | 5 | 1.35 * | 0.90 | 6 |
| ***BRCA1*** | 0.32 | 0.16 | 10 | 0.60 * | 0.29 | 9 |
| ***G6PD*** | 0.31 | 0.27 | 12 | 0.30 | 0.24 | 12 |
| ***IL-15*** | 0.84 | 0.23 | 12 | 0.79 | 0.53 | 14 |
| ***PGC1a*** | 1.14 | 0.67 | 13 | 0.88 | 0.30 | 15 |
| ***SCO2*** | 0.78 | 0.32 | 13 | 0.73 | 0.53 | 14 |
| ***SOD2*** | 0.78 | 0.29 | 13 | 0.74 | 0.38 | 14 |
| ***TP53*** | 0.50 | 0.23 | 13 | 0.54 | 0.29 | 14 |
| ***UCP3*** | 1.01 | 0.78 | 13 | 0.99 | 0.81 | 15 |
| ***VEGFa*** | 0.91 | 0.25 | 13 | 0.90 | 0.34 | 14 |
| ***NR4A1*** | 0.90 | 0.41 | 13 | 0.92 | 0.62 | 14 |
| ***NR4A2*** | 0.68 | 0.36 | 13 | 0.59 | 0.28 | 9 |

**Table e:** Mean MyHC composition as well as MyHC composition for HPF and LPF with regard to MyHC I, MyHC IIa and MyHC IId/x fiber type.

| **Variables [%]** | **Total (n=25)** | | **HPF (n=13)** | | **LPF (n=12)** | |
| --- | --- | --- | --- | --- | --- | --- |
|  | Mean | SD | Mean | SD | Mean | SD |
| **MyHC I** | 17.30 | 5.55 | 17.53 | 5.76 | 17.06 | 5.55 |
| **MyHC IIa** | 36.98 | 4.15 | 36.43 | 4.34 | 37.58 | 4.02 |
| **MyHC IId/x** | 45.71 | 3.39 | 46.03 | 3.76 | 45.37 | 5.14 |
